# The Stock Market Model with Delayed Information Impact from a Socioeconomic View

**DOI:** 10.3390/e23070893

**Published:** 2021-07-14

**Authors:** Zhiting Wang, Guiyuan Shi, Mingsheng Shang, Yuxia Zhang

**Affiliations:** 1 Physics and Photoelectricity School, South China University of Technology, Guangzhou 510640, China; 201820127594@mail.scut.edu.cn; 2 International Academic Center of Complex Systems, Beijing Normal University at Zhuhai, Zhuhai 519087, China; sgy@bnu.edu.cn; 3 Chongqing Institute of Green and Intelligent Technology, Chinese Academy of Sciences, Chongqing 400714, China; msshang@cigit.ac.cn

**Keywords:** econophysics, financial complexity, collective intelligence, emergent property, stock correlation, detrended cross-correlation analysis

## Abstract

Finding the critical factor and possible “Newton’s laws” in financial markets has been an important issue. However, with the development of information and communication technologies, financial models are becoming more realistic but complex, contradicting the objective law “Greatest truths are the simplest.” Therefore, this paper presents an evolutionary model independent of micro features and attempts to discover the most critical factor. In the model, information is the only critical factor, and stock price is the emergence of collective behavior. The statistical properties of the model are significantly similar to the real market. It also explains the correlations of stocks within an industry, which provides a new idea for studying critical factors and core structures in the financial markets.

## 1. Introduction

With the massive use of information and communication technologies, we can collect traceable data from almost anyone. The rise of network science [1] and computational social science [2] have provided opportunities for innovative research in econophysics and sociophysics. In particular, econophysics regards the financial market as a complex system and attempts to depict it more realistically, such as the interactions between investors by network dynamic evolution. Econophysics describes the economic system with many interacting heterogeneous entities (people, firms, institutions, etc.), and expects to find similar laws to the physical system. However, humans are not ideal gas molecules, it is unclear how many and which quantities would be needed for determining and anticipating a given macroscopic, in the sense of collective, observable [3]. Moreover, because human beings are adaptable, the study of economic systems is bound to be a difficult problem.

Researchers have proposed numerous different mechanisms to model the microstructure of financial markets. They pursued the most detailed descriptions, such as creating diverse agents and setting rules for interactions between agents and trading rules. Researchers collected data about investors’ behavior through information technology to deal with the variables of different individuals. But individuals rely on different risk preferences and reference points. Even if we can reasonably describe the behavior of a single individual, we cannot directly generalize to a group. Investors’ decisions in financial markets are not always rational; their buying and selling decisions are affected by emotion, personality, and bias [4,5]. People are different, and they are not rational to some extent. For individuals, faith may be stronger than reason, personal interest may be stronger than the good of the team, etc. Meanwhile, the COVID-19 disease is a new and dreaded event [6], and in the process of keeping the virus under control, people’s cognitive functioning has been enhanced, and their behavior has been changed to some extent. For example, more people are willing to wear a mask after the epidemic outbreak and so on. In the stock market, there are so many unpredictable fluctuations. When new information is generated, what does it mean for the stock market? That would hardly be positive for the stock market because investors are different, and their cognitive processes and cognitive environment are fickle and changeable. In the face of changes in the information environment, different investors have different reaction capacities and speeds. After thinking about the information, even for a specific investor, they will understand the information from a new perspective and form their own judgment slowly. Thus, in a financial system, microstructure models are not enough to consider the variable adaptability of investors.

Although investors are different and unpredictable, research exhibits that pieces of statistical evidence remain stable, accordant to the stability of the statistical properties of particle motion in physics models [7,8]. Therefore, in the studies of financial markets, statistical results of different micro models exhibit universal characteristics. The classical percolation model [9,10,11] simulates herd behavior. For any pair of agents *i* and *j*, they are connected with a probability, and then agent *i* makes the buying or selling decision with another probability. The model explains the power-law distribution of stock price returns appropriately. The two-dimensional Ising model [12] considers investors’ imitation of neighbors, the influence of public information, and personal traits. Here the influence of public information is a Gaussian distribution. The investor’s decision function also has a probability form, and the returns of the model are “fat-tailed” [13,14]. The financial models with network topology [15] also produce the universal characteristics of real stock markets by setting the link probability of nodes and performing decision functions. These models share common features. First, they generate a stock trading environment in the form of probability. Second, investors make buy-sell decisions with probability or decision functions. More details are introduced to depict a more realistic financial market based on these basic models and their common features. Over the past century or so, stock trading information flow has changed from slow to intensive, investors’ literacy from low to high, relationship from simple social relationships to complex social networks. Individual characteristics of investors and the market environment have dramatically changed. Stock trading rules also varied in different countries; for example, China has a 10% price limit [16]. Nevertheless, no matter what changed the environment or rules, it is observed that universal characteristics are robust on different timescales and in different stock markets. Therefore, in the study of the macro laws, statistical properties of the stock market, the critical factor should not be the relationship network of investors, the speed of information flow, or the level of literacy of investors, which researchers want to introduce. On the other hand, collective intelligence results from intelligence, which emerges out of collaboration and coordination of many individual agents [17]. Collective intelligence, which Wooley et al. [18] define as the ability of a group to perform a wide variety of tasks. They studied “collective intelligence” and demonstrated that the critical factor characterizing “collective intelligence” is not the group members’ average or maximum individual intelligence. Here, we view the ability of investors to make buying and selling decisions. Investors gamble in the stock market, where supply and demand determine the stock price, i.e., the result of their behavior is reflected in the price of the stock. Investors’ collective intelligence is the emergence of investors’ collective behavior. In this paper, we abstract all the factors that impact the market to the only value of information. In given information, the behavior of investors emerging with probabilities results in the evolution of stock markets. Here, unlike the micro model that pursues a realistic and detailed structure, we discard individual features and interaction. We present a stock price evolution model with emergence properties in the given information in Section 2 and verify its rationality using real market data in Section 3. We aim to find the critical factor and capture stable macroscopic law in the ever-changing stock market.

The paper is organized as follows: Section 2: A detailed description of the stock market model with delayed information impact. Section 3: Statistical analysis and nonlinear behavior of the proposed model. Section 4: Correlation analysis between stocks in the industry.

## 2. Stock Price Model with Delayed Information Impact

The analysis of financial stock market prices has been found to exhibit some universal characteristics similar to those observed in physical systems with many interacting units, and several microscopic models have been developed to study them. Examples include percolation models, Ising models, network models, and their extensions to social interactions. Though these models are very different, they all can be used to simulate the stock market. Because the simulation results are consistent with the statistical properties of real market price fluctuations, these models may generate the “Newton’s laws” in financial markets. Thus, we aim to find the possible “Newton’s laws” in these models and try to prove it.

The classical percolation model is generated with the connection probability of neighbor nodes. The Ising model is a random field with a probability, and the evolution of the swing is closely related to the structure of space and initial state. The network model is also generated with a probability. We find the common feature that they generate is stock trading in the form of probability.

Mitchell and Mulherin [19] studied the relation between the number of news announcements reported daily by Dow Jones & Company and aggregate measures of securities market activity, including trading volume and market returns. They employed a distinctive proxy for the information, i.e., the number of announcements released daily by Dow Jones & Company. Meanwhile, the social sciences have obtained access to huge datasets based on the internet activity of millions of users all over the world. Among the most frequently utilized providers of data, social media such as Twitter and Facebook and search engines Google and Yahoo play the most important roles. For example, the frequency of searched terms has been shown to provide helpful information for forecasting various phenomena ranging from trading volumes [20] to consumer behavior [21] and finance [22]. In summary, information is too complicated to be considered fully in a theoretical model, let alone delayed information in stock markets. In previous studies, Albers et al. [23] studied “delayed information.” In the paper, the time when relevant information is available and the time that a decision has an effect could be decoupled. Investors might not have access to the latest exchange rates or stock prices. They refer to this as the delayed information model. However, we define a new concept of delayed information here. In the stock market, information comes in various ways and at different influence levels. In general, there is a small amount of super good news and bad news. Most of the news is ordinary. In our model, the influence of information is an abstract concept. The influence of information will last for some time, and the disappearance time of influence will be delayed. This is what we refer to as delayed information.

We propose the stock price model of delayed information impact based on the common feature and abstract information. It includes two components, i.e., the generation and delay of market information and the emergence of collective decision-making in the given information.

### 2.1. Information Generation and Delay

- Suppose the initial stock price is $P_{0}$. The stock market environment is fickle daily and is influenced by a series of stochastic events, including supply and demand, macroeconomic, political factors, corporate finances and performance, market sentiment, etc. We coarse-grain all the stochastic events by information into just a single influence value. The impact of information is an abstract concept, which is a random variable that is normally distributed with mean 0 and standard deviation $\sigma_{1}$, here $\sigma_{1}=\lambda P_{0}$. Any theoretical normal distribution has a maximum of infinity and a minimum of minus infinity. There is an infinite range. In our model, the impact of information is normally distributed, and it must be finite. Thus, there must be a truncation. The truncation interval should be large enough and reasonable. The information has an impact on the stock price, so the truncation interval has a relation with the stock price *P*_0_. It cannot stand alone. Here, considering the extreme cases (terrible information, great information), we set the truncation interval to be$\;\left[-4\sigma_{1},+4\sigma_{1}\right]$. New information sequence $I_{t}$ can be obtained by random sampling from the truncated Gaussian distribution.

- In the stock market, the influence of information is in a state of change and eventually disappears. Thus, we introduce the delayed information. The progress of influence disappearance is a different matter from the memory deterioration. That the influence of information eventually disappears does not mean that the people forget the information; it is just that the information is a dead issue. Considering that significant events have a sustained impact on the investors, and the impact strength of the information will delay over time, we assume that the information influence $I_{t}$ delays linearly with time simply, and the information influence after the *i*-th day $I_{i}^{\prime }$ is expressed as
  (1) $$I_{i}^{\prime }=\left\{\begin{array}{c} I_{t}-ai,I_{t}>0\\ I_{t}+ai,I_{t}<0 \end{array}\right.$$
  where a is the delay coefficient.

### 2.2. Stock Price Evolution Process

The given information determines the theoretical stock price $P_{t}^{\prime }$.

(2) $$P_{t}^{\prime }=P_{t-1}+I_{t}+\sum_{i=0}^{i=t-1}I_{i}^{\prime }$$

Investors participate in the game and make decisions based on the given information. Their collective behaviors result in actual stock prices. As the investors vary from radicals or conservatives, daredevils, or followers, etc., statistical properties of the final actual stock price series are stable in the ever-changing stock market. The actual stock price $P_{t}$ in day *t* has emergence properties of collective intelligence, which is a random sampling from a truncated Gaussian distribution $P_{t}\sim N\left(P_{t}^{\prime },\sigma_{2}^{2}\right)$. As the price fluctuation is related to the information, here $\sigma_{2}=\frac{1}{3}\times \left|P_{t}^{\prime }-P_{t-1}\right|$. Considering the extremes, we set the truncation interval as $\left[-4\sigma_{2},+4\sigma_{2}\right]$.

**Figure 1:**
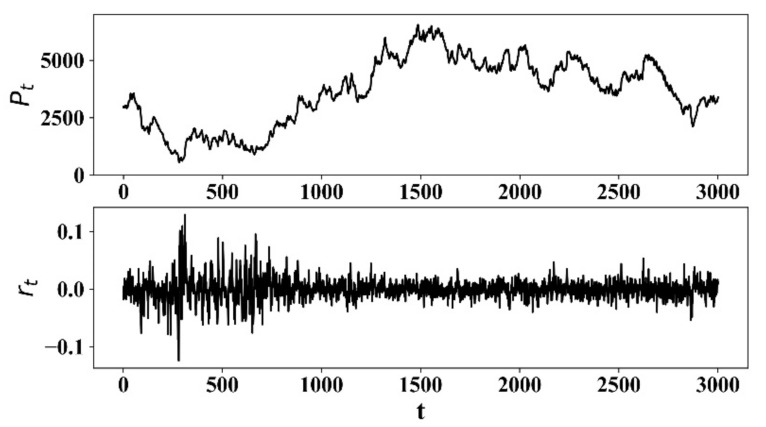
Stock price series of the proposed model and its corresponding return.

Figure 1 shows the simulated stock price series $P_{t}$ and the corresponding return series $r_{t}$,$\;P_{0}=3000,\sigma_{1}=20,a=5$. In Figure 1, volatility clustering is easily observable. High-volatility tends to follow high-volatility, and low-volatility tends to follow low-volatility.

## 3. Descriptive Statistics and Nonlinear Behavior Analysis

This section discusses the stock price model’s descriptive statistics and nonlinear behavior with delayed information impact and verifies the simulation results with the real stock market. We use real daily closing price data from 1 January 2010 to 3 December 2020 (*T* ≈ 2700), including the SSE (Shanghai Composite Index), SZES (Shenzhen Stock Exchange Index), and S&P500 (S&P 500 Index) (https://finance.yahoo.com, 3 May 2021). The simulated data length *T* = 3000 matches with the real data (*T* ≈ 2700).

### 3.1. Descriptive Statistics of Returns

**Figure 2:**
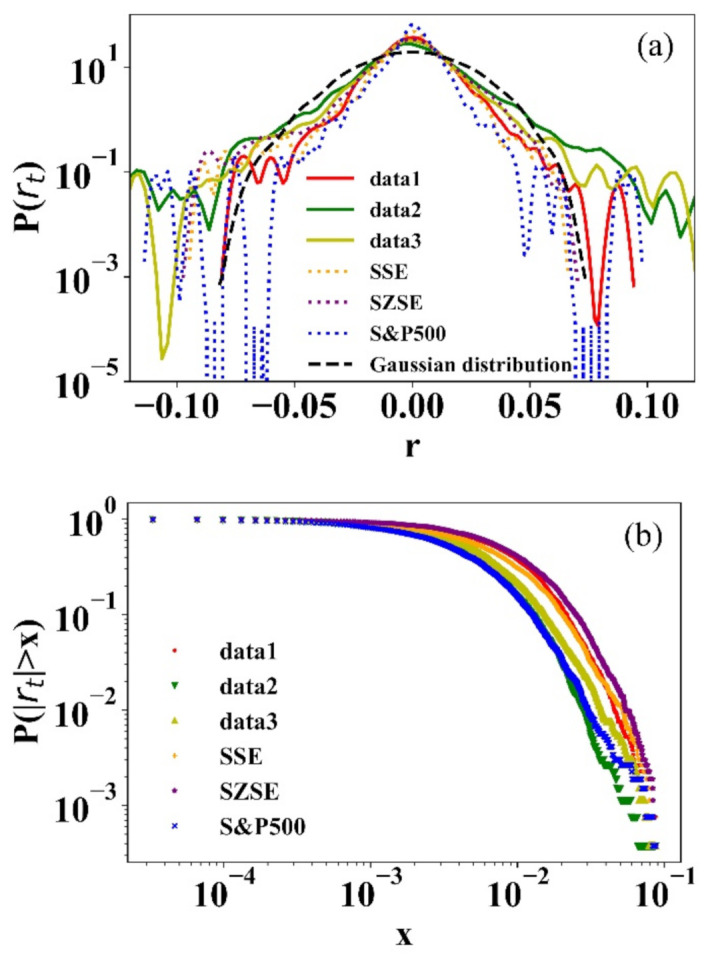
(**a**) The probability density distributions of simulated and empirical returns (semi-log); (**b**) The cumulative distributions of simulated and empirical returns (log-log).

The “Fat-tailed” characteristic of returns has been verified in extensive empirical studies [24,25,26]. It is an important criterion for the reasonableness of price dynamics in the stock model research. Here, the definition of price return is $r_{t}=lnP_{t}-lnP_{t-1}$ [27]. The probability density distributions of three simulated and real market returns are shown in Figure 2a. Simulated and real return distributions are almost identical. Compared to the Gaussian distribution, they both exhibit distinct “fat-tailed” characteristics. Table 1 shows the statistics: mean, standard deviation, maximum, minimum, skew, kurtosis, the results of Kolmogorov–Smirnov test (K-S test) and power-law fit, where the kurtosis of all returns is larger than three that is the kurtosis of the Gaussian distribution [28]. In the K-S test, all *p*-values are very small, and all the H-values are 1, so we reject the null hypothesis that the distribution follows the Gaussian distribution at a 5% significance level. Figure 2b shows that the cumulative probability distributions of simulated and real market returns follow power-law distribution $\;P\left(\left|r_{t}\right|>x\right)\sim x^{-\alpha }$, α is the power-law exponent. The corresponding power-law exponent values in Table 1 approximately equal to 3, it obeys the “Inverse cubic law” [29].

### 3.2. Nonlinear Statistical Analysis of Returns

Some studies have investigated the nonlinear properties of financial markets [30,31,32]. Hsieh [30] discussed some of the methodological issues in detecting chaotic and nonlinear behavior. Alves et al. [31] focused on the Dow Jones Index to determine the chaotic dynamics. Zhu et al. [32] revealed the long-term memory of financial time series. Here, we compare the nonlinear behavior of the simulated return series with the real market series.

#### 3.2.1. Correlation Dimension Analysis

The correlation dimension method measures the complexity of dynamical systems that distinguishes deterministic systems (including low-dimensional chaos) and stochastic systems [33]. According to the method of Grassberger et al. [34], the correlation dimension can be calculated when the appropriate embedding dimension *m* and time lag *τ* are selected for the phase space reconstruction. For an *m*-dimensional phase space, the correlation integral *C(r)* is calculated by

(3) $$C\left(r\right)=\underset{N\to \infty }{lim}\frac{2}{N\left(N-1\right)}\overset{N}{\underset{i,j=1,i\neq j}{\sum }}\Theta \left(r-\left|X_{i}-X_{j}\right|\right)$$

where Θ is the step function. The appropriate choice of *r* enables the correlation dimension of the system *D* to describe as

(4) $$D=\underset{r\to 0}{lim}\frac{{log}_{2}C\left(r\right)}{{log}_{2}r}$$

**Figure 3:**
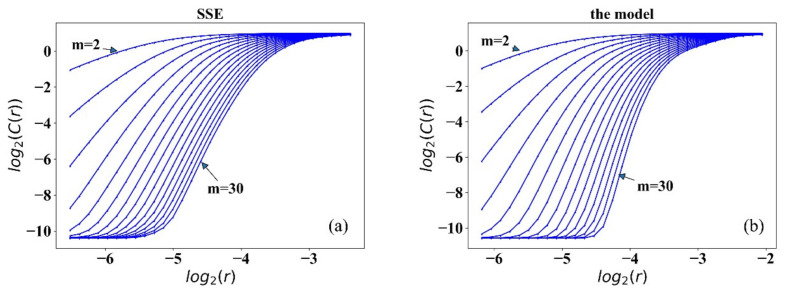
Correlation integral results of return series from SSE (**a**), the model (**b**).

**Figure 4:**
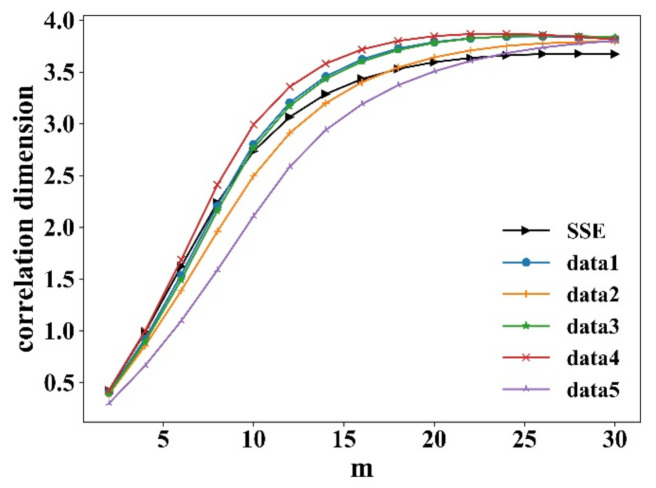
Correlation dimension of returns from SSE and five simulated data.

A common method is to fit the ${log}_{2}C\left(r\right)$ and ${log}_{2}r$ using least squares, and the slope is the correlation dimension *D*. For random sequences, *D* increases linearly with the embedding dimension *m* with no saturation. While for deterministic chaotic sequences, *D* increases with *m* to a certain position to reach saturation, and the saturation *m* is the correlation dimension *D* of the time series attractor. Figure 3 shows the correlation integral ${log}_{2}C\left(r\right)$ and ${log}_{2}r$ in different embedding dimensions *m*. Figure 4 shows the correlation dimension. It is observed that all correlation dimensions increase with *m* and reach saturation at a certain position. It can be seen that all the returns have deterministic noise, which means the systems are chaotic. The simulated data from the proposed model coincide with the real market data.

#### 3.2.2. Lyapunov Exponent Analysis, Sample Entropy Analysis, and Hurst Exponent

We further compare the nonlinear behavior of simulated and empirical rates of return in this section. The maximal Lyapunov exponent (MLE) determines the predictability of a dynamical system. A positive MLE is usually taken as an indication that the system is chaotic. Consequently, any system with MLE > 0 is considered to be chaotic. We calculate the MLE of each stock price series using the algorithm of Rosenstein et al. [35]. In Table 2, the simulated and real returns have similar positive MLE, and indicate they are not totally stochastic. They have a similar chaotic property to some extent.

Hurst exponent is used as a measure of the “long memory” of a time series, which measures how the range of fluctuations in a time series varies over time. H ranges between 0 and 1 (excluding 0 and 1). Where H = 0.5, the time series indicates a completely uncorrelated series. When H > 0.5, the time series has long-term memory, and when H < 0.5, the time series has inverse persistence, it exhibits stronger fluctuations than totally random. We calculate the Hurst exponent by the rescaled range analysis [36]. In Table 2, the Hurst exponent is slightly larger than 0.5, which means that the simulated and real returns have similar long-term memory.

Sample entropy is a measure of the complexity of time series. The smaller the sample entropy, the higher the sequence self-similarity; the larger sample entropy, the more complex the sample sequence. We calculate the sample entropy method following Richman et al. [37]. In Table 2, the simulated and real returns have similar sample entropy values that indicate their similar complexity.

## 4. Correlation Analysis of Stocks

Portfolio theory is a framework for assembling a portfolio of assets such that the expected return is maximized and the level of risk is minimized. Investors can reduce risk by holding a portfolio of stocks that are not perfectly positively correlated. Diversification can help to construct optimal investment portfolios. Charu et al. [38] use mutual information for measuring stock correlations and construct the stock network. Sun et al. [39] applied DCCA coefficients to construct the correlation matrix of assets. Thus, the correlation between stocks is an important criterion to weigh the correlation of stock market risk level and portfolio rationality. Studies on the properties of stock correlation show that the stronger correlations between stocks are, the higher risk in the corresponding asset portfolio [40]. Usually, stocks belonging to the same industry are more correlated because they are influenced by the same external information, including natural climate, macro policies, raw materials, and other factors [41]. The stocks in an industry have strong positive correlations and risky portfolios, so sound investments usually cover different industries. In our model, stock rises or falls are affected by external information; thus, the model can be considered to study the correlation between stocks.

This section investigates the correlation of stock returns within per industry in China using the detrended cross-correlation analysis (DCCA) [42,43] and calculates their distributions. The DCCA coefficient measures the correlation level between non-stationary series such as financial series. ρ is the DCCA coefficient, −1≤ρ≤1. ρ=1 indicates that two time series are perfectly correlated; ρ=−1 indicates that the two time series are perfectly anti-correlated; ρ=0 indicates that the two time series are uncorrelated processes. There are 28 industries in the Shenwan Industry Classification Standard. We selected 16 industries from 1 January 2016 to 10 December 2020 $\left(T\approx 1200\right)$, which contain a sufficient number of stocks (the number of stocks N>30). We then simulated stock data in an industry: As the initial stock price is the same, to avoid the sensitivity to initial conditions, we selected the data from 6000 to 7500 steps in the simulation (*T* = 1500), then we obtained 100 stocks under the same historical information series.

**Figure 5:**
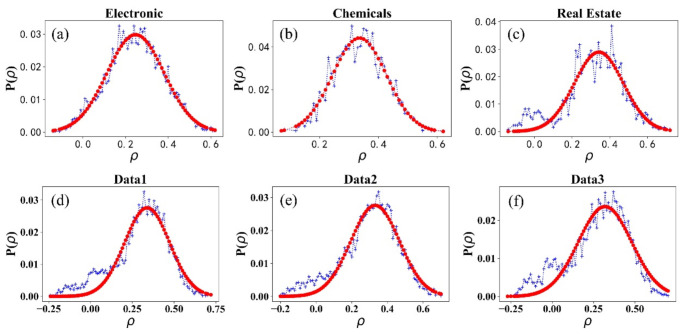
The distribution of ρ from Chemicals (**a**), Real Estate (**b**), Electronics (**c**), and three simulated data (**d**–**f**).

**Figure 6:**
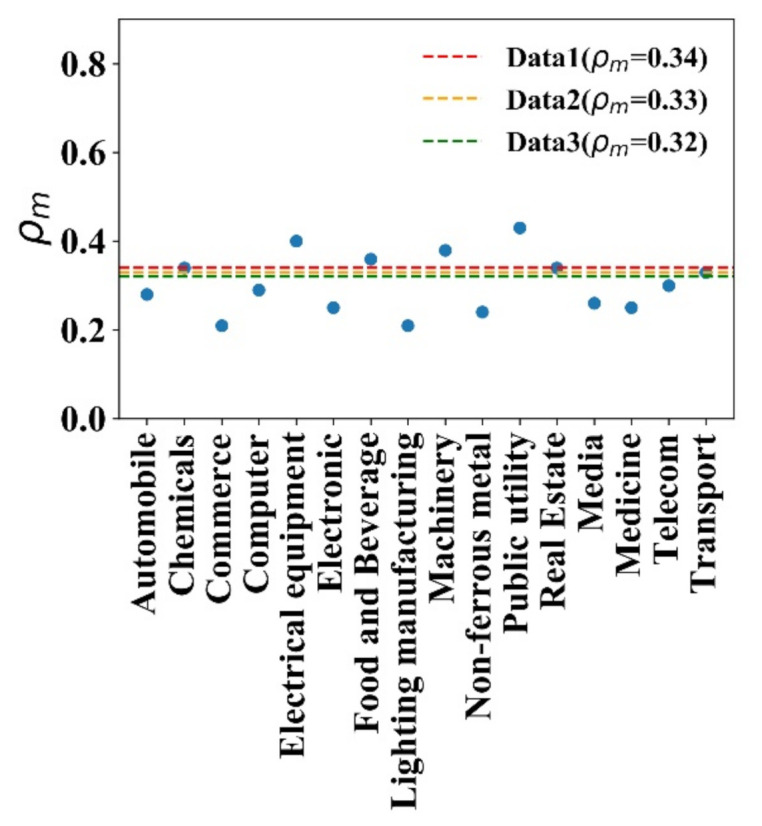
The $\rho_{m}$ of stocks in 16 industries and three simulated data.

Figure 5 shows the distribution of the correlation of stock returns within an industry. Figure 5a–c are three empirical data examples, and Figure 5d–f are three simulated ones that are generated in different historical information series. It can be seen in Figure 5 that ρ distributions within each of the 16 industries show a regular single-peaked distribution. The most probable correlation coefficients $\rho_{m}$ are around 0.3, which indicates that the model is consistent with the real market, and most stocks have weak positive correlations within an industry. Figure 6 shows the most probable correlation coefficients $\rho_{m}$ within the 16 industries and the three simulated data. The three simulated data peaks are 0.34, 0.33, and 0.32; all are lying within the peak range from 0.21 to 0.43 in the real market. Moreover, since each set of simulated data is generated in given the same historical information series, there is probable that the stock market evolution will recur when there is similar information series. In our model, the correlation of the simulated stock with the same historical information can be analogized statistically to the correlation of the stocks within China’s industry. It is a supplement method of stock correlation research that helps investors obtain a better portfolio strategy.

## 5. Conclusions

“Greatest truths are the simplest” is an objective law. The principles also apply to the stock market. With the development of the stock market, the spread of information is faster. It is easier to get information, the literacy level of the investors has improved. They are closer to each other; their relationships are more complicated than ever, society’s wealth has increased, etc. The empirical studies show that no matter how the stock market environment changes, the universal characteristics (the crashes, the skewed distributions with specific kurtosis values, the fat tails, etc.) remain stable. It means that the “Greatest truths in stock market remain stable.” In this paper, we aim to find the “Greatest truths in the stock market.”

We analyze three typical models (the percolation model, Ising model, and network topology financial model) and their extensions that are used for stock market research. We find that these models can represent the universal characteristics successfully. It means that these models should contain the “Greatest truths in the stock market.” We find that “they generate a stock trading in the form of probability.”

The stock market environment is variable daily and is influenced by a series of stochastic events (supply and demand, macroeconomic, political factors, corporate finances and performance, market sentiment, etc.). We coarse-grain all the stochastic events by information just a single influence value. The information can influence investors’ performance. The stock price is the result of all investors’ performance. We model the progress in probability and find that it can represent the universal characteristics.

Our model is based on the idea of “Greatest truths in the stock market.” Our results suggest that the investors’ individual characteristic is not the critical factor; the stock market’s micro-specialties are not the greatest truths. In the stock market, the critical factor is information, and the stock price is the emergence of collective performance of all investors. Besides, the model can generate different stock price series in the same historical information, analogous to the stocks in the same industry. Similar single-peaked distribution proving that the model can be effectively used in stock correlation research and history recur rules. It opens a new way of selecting rational portfolios, complementing current industry correlation research methods, and providing theoretical support. The paper provides a helpful framework for understanding stock price evolution through the emergence of collective performance. We find the possible critical factor and the essence of the financial market at a macro level.

## Figures and Tables

**Table 1:** Descriptive statistics, power-law fit, and K-S test of returns.

Data	Mean	Std	Max	Min	Skew	Kurtosis	K-S Test		α
							*p*-Value	H	
$x_{1}$	0.00004	0.0172	0.1294	−0.1240	0.2987	6.6543	8.1208 × 10^−9^	1	3.5784
$x_{2}$	−0.00002	0.0217	0.1601	−0.2125	−0.3848	9.3611	1.8554 × 10^−10^	1	4.0968
$x_{3}$	0.00005	0.0182	0.1520	−0.1367	−0.0234	8.3799	4.2418 × 10^−10^	1	3.8109
S&P500	0.00004	0.0111	0.0934	−0.1066	−0.9710	15.2922	4.0739 × 10^−18^	1	3.4624
SSE	0.00002	0.0136	0.0060	−0.0887	−0.8969	6.1958	1.6704 × 10^−10^	1	3.5277
SZSE	0.00001	0.0164	0.0625	−0.0895	−0.7368	3.7987	5.8053 × 10^−7^	1	3.4777

**Table 2:** The maximum Lyapunov exponent (m = 10), Sample Entropy (m = 2) and Hurst exponent of returns from the model and empirical market.

Data	MLE	Sample Entropy	Hurst Exponent
Data1	0.0778	1.7497	0.6281
Data2	0.0762	1.6832	0.6364
Data3	0.0773	1.7033	0.6478
Data4	0.0757	1.7401	0.6152
Data5	0.0575	1.4901	0.5840
SSE	0.0628	1.7889	0.5238
SZSE	0.0842	1.8750	0.5176
S&P500	0.0639	1.4902	0.5022

## Data Availability

Data sharing not applicable.
